# Boylston Medical Prize Questions

**Published:** 1842-09

**Authors:** 


*Boylston Medical Prize Questions.*—We thank our friend, the editor of the "Boston Medical and Surgical Journal," for reminding us of a duty which we ought long since to have performed, namely, to have notified the readers of the "American Journal and Library of Dental Science," that among the prize questions for 1843 is one on "the structure and diseases of the teeth, with a numerical solution of the question, can caries of the teeth be retarded by mechanical processes?" We saw the advertisement more than a year ago, and intended at the time to have copied it into our Journal, but it afterwards, in some way or other, escaped our attention; but we now with great pleasure give place to it.

"The Boylston Medical Committee, appointed by the President and Fellows of Harvard University, consists of the following physicians:—John C. Warren, M. D., George C. Shattuck, M. D., Jacob Bigelow, M. D., Walter Channing, M. D., George Hayward, M. D., John Randall, M. D., Enoch Hale, M. D., John Ware, M. D., Edward Reynolds, M. D. At the annual meeting of the Committee, August 3, 1842, the Boylston Premium of fifty dollars value was awarded to William A. Davis, M. D., of Springfield, Massachusetts, for the best dissertation on "The diseases of the kidney; and the changes which occur in the appearance and composition of the urine in health and disease."

"The questions for 1843 are—

"1st. The best method of warming and ventilating rooms for preventing and curing disease.

"2d. The structure and diseases of the teeth, with a numerical solution of the question, can caries of the teeth be retarded by mechanical processes?

"Dissertations on these subjects must be transmitted, post paid, to John C. Warren, M. D., Boston, on or before the first Wednesday of April, 1843.

"The following questions are proposed for 1844:

"1st. To what extent is the human system protected from smallpox by inoculation with the cowpox? Is the protection increased by re-vaccination, and if so, under what circumstances?

"2d. In what cases, and to what extent, is the division of muscles, tendons, or other parts, proper for the relief of deformity or lameness?

"Dissertations on these questions must be transmitted as above, on or before the first Wednesday of April, 1844.

"The author of the best dissertation on either of the above subjects, will be entitled to a premium of fifty dollars, or a gold medal of that value, at his option.

"Each dissertation must be accompanied by a sealed packet, on which shall be written some device or sentence, and within shall be enclosed the author's name and residence. The same device or sentence is to be written on the dissertation to which the packet is attached.

"All unsuccessful dissertations are deposited with the Secretary, from whom they may be obtained, if called for within one year after they have been received.

"By an order adopted in 1826, the Secretary was directed to publish annually the following votes, viz:

"1st. That the Board do not consider themselves as approving the doctrines contained in any of the dissertations to which the premiums may be adjudged.

"2d. That in case of the publication of a successful dissertation, the author be considered as bound to print the above vote in connection therewith.

*Boston, August* 4, 1842. ENOCH HALE, *Secretary.*"

An opportunity is here offered for some one of our professional brethren to distinguish himself, and we hope it will not be allowed to pass unimproved.

